# The hypothalamus and periaqueductal gray are the sources of dopamine fibers in the paraventricular nucleus of the thalamus in the rat

**DOI:** 10.3389/fnana.2014.00136

**Published:** 2014-11-20

**Authors:** Sa Li, Yuxiu Shi, Gilbert J. Kirouac

**Affiliations:** ^1^PTSD Laboratory, Department of Histology and Embryology, Institute of Pathology and Pathophysiology, China Medical UniversityShenyang, China; ^2^Department of Oral Biology, Faculty of Dentistry, University of ManitobaWinnipeg, MB, Canada; ^3^Department of Psychiatry, Faculty of Medicine, University of ManitobaWinnipeg, MB, Canada

**Keywords:** paraventricular nucleus, dopamine, ventral tegmental area, hypothalamus, thalamus

## Abstract

The paraventricular nucleus of the thalamus (PVT) sends a very dense projection to the nucleus accumbens. This area of the striatum plays a key role in motivation and recent experimental evidence indicates that the PVT may have a similar function. It is well known that a dopaminergic projection from the ventral tegmental area (VTA) to the nucleus accumbens is a key regulator of motivation and reward-related behavior. Dopamine (DA) fibers have also been localized in the PVT but the source of these fibers in the rat has not been unequivocally identified. The present study was done to re-examine this question. Small iontophoretic injections of cholera toxin B (CTb) were made in the PVT to retrogradely label tyrosine hydroxylase (TH) neurons. Neurons that were double-labeled for TH/CTb were found scattered in DA cell groups of the hypothalamus (ventrorostral A10, A11, A13, A15 DA cell groups) and the midbrain (dorsocaudal A10 embedded in the periaqueductal gray). In contrast, double-labeled neurons were absent in the retrorubral field (A8), substantia nigra (A9) and VTA (A10) of the midbrain. We conclude that DA fibers in the PVT do not originate from VTA but from a heterogeneous population of DA neurons located in the hypothalamus and periaqueductal gray.

## Introduction

The paraventricular nucleus of the thalamus (PVT) is a member of a group of midline and intralaminar thalamic nuclei that are believed to be involved in cognition, attention and arousal (Groenewegen and Berendse, 1994; Van der Werf et al., 2002). Each member of this group has circumscribed projections to unique regions of the cerebral cortex and the striatum. This anatomical arrangement has led to the view that different members of the midline and intralaminar nuclei are part of functionally distinct circuits with specialized functions (Groenewegen and Berendse, 1994; Van der Werf et al., 2002). The PVT is of special interest because of its dense projection to the nucleus accumbens of the ventral striatum (Li and Kirouac, 2008; Vertes and Hoover, 2008), an area of the brain closely linked to the regulation of reward and motivation (Nicola, 2007; Humphries and Prescott, 2010). In addition, the PVT projects to all the major sources of inputs to the nucleus accumbens including the medial prefrontal cortex (ventral prelimbic and infralimbic cortices), ventral subiculum of the hippocampus, and the basolateral nucleus of the amygdala (Groenewegen et al., 1987; Berendse and Groenewegen, 1990; Berendse et al., 1992; Brog et al., 1993; Wright and Groenewegen, 1995; Wright et al., 1996).

Recent experimental evidence supports a role for the PVT in drug seeking and reward-related behaviors (Martin-Fardon and Boutrel, 2012; James and Dayas, 2013; Matzeu et al., 2014). For instance, an increase in cFos has been observed in the PVT following the re-exposure of rodents to the context or cues associated with cocaine administrations (Brown et al., 1992; Franklin and Druhan, 2000; James et al., 2011). Inactivation of the PVT was also reported to attenuate cocaine-prime induced reinstatement of self-administration as well as cocaine induced conditioned place preference (James et al., 2010; Browning et al., 2014). Similarly, an increase in cFos was found in the PVT following context- and cue-induced reinstatement of alcohol seeking in rodents (Wedzony et al., 2003; Dayas et al., 2008; Perry and Mcnally, 2013) while inactivation of the PVT was reported to attenuate context-induced reinstatement of ethanol (Hamlin et al., 2009; Marchant et al., 2010). Finally, evidence in support of a role for the PVT in food reward is provided by studies showing an increase in cFos in the PVT following exposure to cues that predict a sucrose reward (Igelstrom et al., 2010; Flagel et al., 2011).

Dopamine (DA) neurons in the ventral tegmental area (VTA) and their projections to the nucleus accumbens have been implicated in reward and motivation (Pennartz et al., 1994; Ikemoto and Panksepp, 1999; Schultz, 2000; Salamone et al., 2005; Ikemoto, 2007). The PVT has also been shown to contain fibers that stain for tyrosine hydroxylase (TH), the rate limiting enzyme involves in the synthesis of DA (Takada et al., 1990; Otake and Ruggiero, 1995). In addition, the PVT contains fibers immunopositive for DA as well as the DA transporter indicating that some of the TH fibers in that area of the thalamus are dopaminergic fibers (Groenewegen, 1988; García-Cabezas et al., 2009). Indeed, the PVT was reported to contain a more notable plexus of immunopositive DA fibers than other dorsal midline thalamic nuclei (Groenewegen, 1988). Consequently, it is possible that the PVT is part of the neural circuitry that mediates DA’s motivational functions (VTA-PVT-nucleus accumbens). However, the two studies that have examined the source of DA fibers in the PVT in the rat have come to different conclusions with one study finding evidence that the VTA provided the input (Takada et al., 1990) while the other reporting that the hypothalamus was the source (Otake and Ruggiero, 1995). Part of this discrepancy may be due to differences in the placement of retrograde tracers in the midline thalamus. The interpretation of these two studies is also limited by the fact that the location of the injections is not clearly shown.

It is important that the sources of DA fibers in the PVT be unequivocally identified to better understand the potential involvement of the PVT in DA-mediated behaviors. In this study, iontophoretic injections of the retrograde tracer cholera toxin B (CTb) were made in the anterior and posterior segments of the PVT (referred to as the aPVT and pPVT, respectively). The location of CTb and TH double-labeled cells were assessed in areas of the brain known to contain DA neurons.

## Materials and methods

### Animals

Male Sprague–Dawley rats weighing 210 ± 10 g (*n* = 8; University of Manitoba vivarium) were used for this study. Animals were housed on a 12/12-h light/dark cycle with food and water freely available. The experimental procedures were in compliance with the Canadian Council on Animal Care and the experimental protocol was approved by Research Ethics Review Board of the University of Manitoba.

### Retrograde tracing experiment

Rats were anesthetized with a mixture of ketamine (Ketalean Hydrochloride 100 mg/ml, 100 mg/kg, i.p.) and xylazine (Rompun 20 mg/ml, 10 mg/kg, i.p.) and given supplementary doses if necessary. Animals were placed in a Stoelting stereotaxic frame and a hand drill was used to expose the brain surface above the target sites. Iontophoretic injections of 0.5% CTb (List Biologicals, Campbell, CA, USA), which was dissolved in 0.01 M phosphate buffer (PB; pH 7.4), were done by applying a positive current of 1.0–2.5 μA (200 ms pulses at 2 Hz for 15 min) through a chlorinated silver wire placed in a glass pipette (approximately 7 μm diameter tip). The coordinates used for injecting CTb were as follows: aPVT at 1.5 mm posterior, 1.0 mm lateral, 5.0 mm ventral, 10° angle toward the middle; and pPVT at 3.0 mm posterior, 0.7 mm lateral, 5.0 mm ventral, 10° angle toward the middle (all coordinates are relative to bregma and the dural surface of the brain with the incisor bar at 3.3 mm below intra-aural line). After the scalp incisions were sutured, rats were returned to their home cages for recovery. The analgesic Carprofen (5 mg/kg, s.c.) was given after surgery and 12 h later. After a 10- to 12-day postoperative survival, rats were deeply anesthetized with 10% chloral hydrate and transcardially perfused with 150 ml heparinized saline followed by 400–500 ml ice-cold 4% paraformaldehyde in 0.1 M PB (pH 7.4). The brains were removed, post-fixed in the same fixative for 1–2 h, and cryoprotected in graded sucrose concentrations (10 and 20% w/v) over 2 days at 4°C. Sections of the brain were taken at 50 μm and placed in 0.1 M phosphate-buffered saline (PBS; pH 7.4) with 0.1% sodium azide and stored at 4°C until the time of the immunoreaction.

### Immunohistochemistry

All immunohistochemical reactions were carried out at room temperature on free-floating sections. Primary and secondary antibodies were diluted in blocking solution containing 0.1% sodium azide, 5% normal donkey serum, and 0.3% Triton X-100 in PBS. Every 4th sections were pre-incubated in the blocking solution for 1 h at room temperature, and then transferred to a cocktail of primary antibodies containing goat anti-CTb (1:10,000; List Biologicals; cat. #703, lot 7032H) and monoclonal mouse anti-tyrosine hydroxylase (TH; 1:100,000; Sigma; cat. #T2928; clone TH-16) and incubated overnight. After 3 rinses, sections were transferred into a biotinylated donkey anti-goat antibody solution (1:500; Jackson Immunoresearch, West Grove, PA, USA) for 1 h. Sections were rinsed again and then exposed to an avidin–biotin complex (Elite ABC Kit; Vector Laboratories, Burlingame, CA, USA) for 60 min. After a few more rinses, the tissue was reacted for 3–5 min with diaminobenzidine (DAB) with nickel intensification (Vector DAB Kit) to produce black CTb labeling. The brain sections were then rinsed in PBS and incubated in biotinylated donkey anti-mouse antiserum (1:500; Jackson Immunoresearch) for 1 h. Sections were rinsed and incubated in ABC for 60 min, rinsed, and reacted for 1–2 min with DAB without nickel intensification to produce brown TH cell labeling. The DAB reaction was terminated by rinsing in PBS before the sections were mounted onto gelatin coated slides and coverslipped.

The antibodies used for these experiments have been previously characterized. The CTb antibody does not bind to any endogenous epitopes in the rat brain (Luppi et al., 1990). The monoclonal TH antibody was produced from the TH-16 hybridoma and purified rat TH was used as the immunogen to produce this antibody. This TH antibody recognizes a specific epitope present on the N-terminal region of rodent TH (technical information provided by manufacturer). Furthermore, this antibody produced the same staining pattern in the rat brain as previously reported (Lindvall et al., 1983; Hökfelt et al., 1984) and negative controls (leaving out the primary antibody) were used to establish specificity of the TH staining.

### Analysis

Brain sections were examined with an Olympus BX51 microscope and only the cases with injections that resulted in a dense core of CTb confined to the PVT were used for the present study. Since the purpose of the present study was to examine the source of DA innervation to the PVT, only areas of the hypothalamus and brainstem containing the DA cell groups were examined for the presence of TH/CTb double-labeled neurons. Neurons expressing DA form a continuum that extends from the periaqueductal gray to the preoptic area of the hypothalamus. This continuum has been subdivided into different groups based on earlier classification (Dahlstroem and Fuxe, 1964; Léger et al., 2010): A8 (retrorubral field of the caudal mesencephalic reticular formation); A9 (substantia nigra pars compacta and lateralis); A10 (ventral tegmental area); A10 dorsocaudal (A10dc, periaqueductal gray matter); A10 ventrorostral (A10vr, cells anterior to the VTA in the supramammillary regon); A11 (from the rostral portion of the periaqueductal gray to the posterior hypothalamus adjacent to the mammillothalamic tract); A12 (arcuate nucleus); A13 (zona incerta region); A14 (along the third ventricle in the rostral hypothalamus including the posterior part of the paraventricular hypothalamamic nucleus); and the A15 (anterior hypothalamus region above the optic chiasm/suproptic nucleus and ventral to the anterior commissure). Every section was examined at high magnification for the presence of CTb granules in TH positive neurons. A figure of a representative case showing TH and TH/CTb-double labeled neurons was produced using Adobe Illustrator (CS4) by digitally superimposing photographed images of the areas in question with digital drawings modified from a stereotaxic atlas (Paxinos and Watson, 2009). Examples of CTb- and TH-positive neurons were photographed using a digital camera (Spot RT Slicer; Diagnostic Instruments, Sterling Heights, MI) mounted on the microscope. The calibration bars were inserted with the Spot software (version 3.2; Diagnostic Instruments) and images were transferred to Adobe Photoshop (CS4) to optimize light and contrast levels.

## Results

### Injections

**Figure 1:**
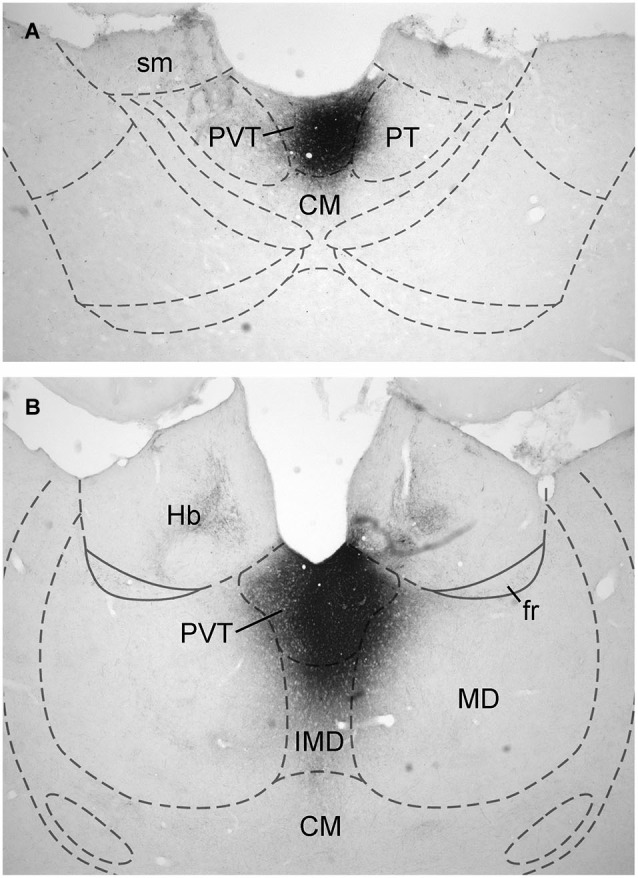
**Examples of injection sites**. Digital image examples of cholera toxin B injection in the anterior **(A)** and posterior **(B)** aspects of the paraventricular nucleus of the thalamus. For abbreviations, see list.

**Figure 2:**
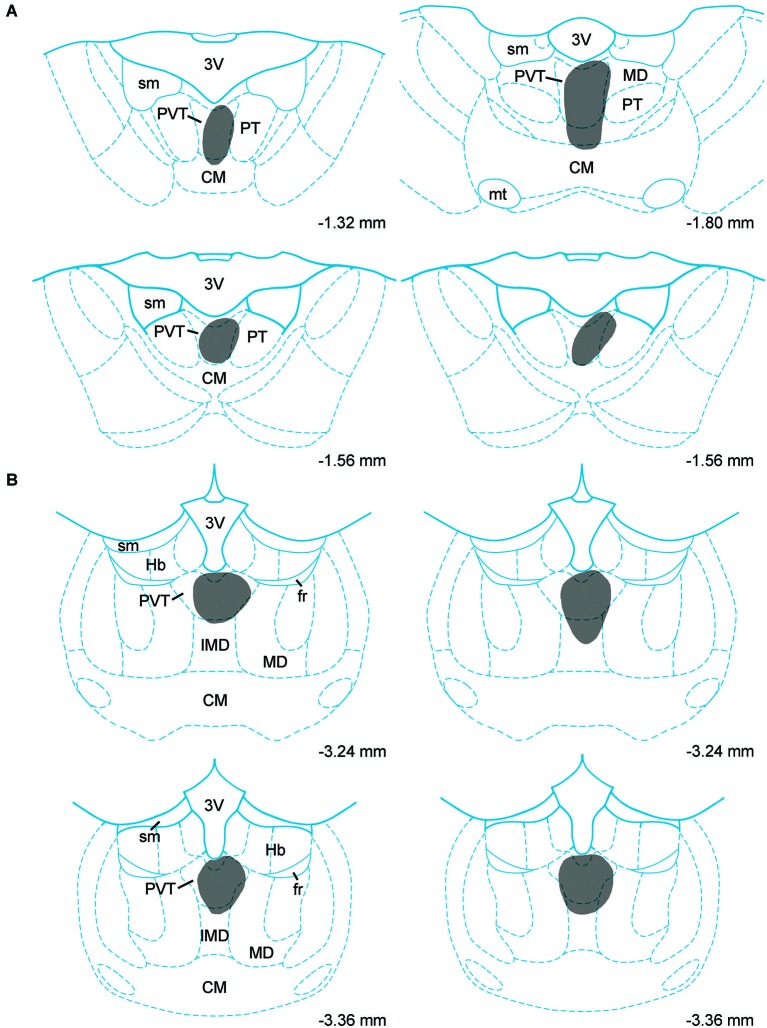
**Injection sites**. Schematic representation of cholera toxin B injections in the anterior **(A)** and posterior **(B)** aspects of the paraventricular nucleus of the thalamus. Numbers on the bottom of each drawing represent the distance from bregma. For abbreviations, see list.

Data on the sources of DA fibers to the PVT were obtained from cases that had CTb injections that were almost entirely restricted to either the aPVT (*n* = 4; Figure 1A) or pPVT (*n* = 4; Figure 1B). Figure 2 shows the location of the dense CTb core of each injection in relation to the PVT and other midline thalamic nuclei. In most cases, the dense core (area likely to result in retrograde transport) covered a large part of the PVT with a very minor involvement of the mediodorsal, paratenial, centromedial, or intermediodorsal nuclei. The PVT extends the entire length of the thalamus and the extent of the injection core was restricted to only a portion of the aPVT or pPVT. This may produce a gross underestimation of DA neurons likely to supply afferents to the PVT. The approach of using small injections was necessary since other tracing studies have shown that the mediodorsal nucleus and habenular complex located immediately adjacent to the PVT are innervated by DA neurons in the VTA (Groenewegen, 1988; Gruber et al., 2007). Large injections of CTb in the dorsal thalamus could have led to incorrect conclusions about the sources of DA afferents to the PVT.

### Pattern of retrograde labeling

**Figure 3:**
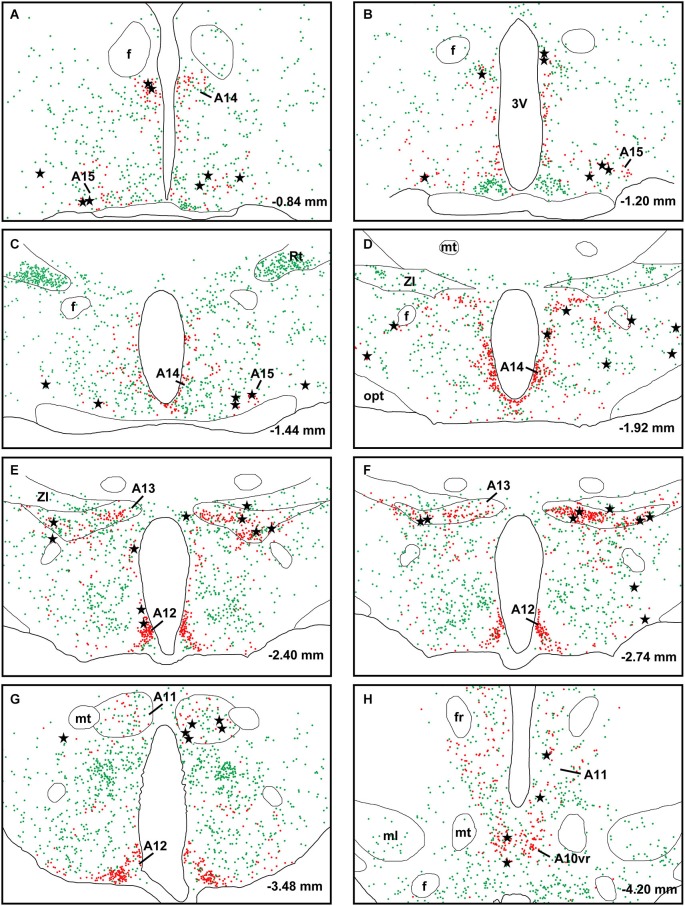
**Retrograde labeling in the hypothalamus**. The rostrocaudal sequence of sections are labeled from **(A–H)**. Drawings of coronal sections with major dopamine cell groups illustrating cells that were immunopositive for tyrosine hydroxylase (TH; red) and cholera toxin B (CTb; green) after an injection of CTb into the posterior part of the paraventricular nucleus of the thalamus. The location of TH/CTb double-labeled neurons are shown as black stars. Numbers on the bottom right of each drawing represent the distance from bregma. For abbreviations, see list.

**Figure 4:**
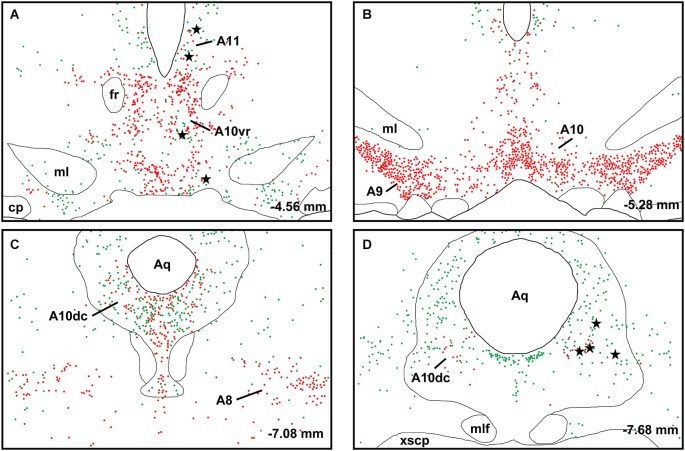
**Retrograde labeling in midbrain**. The rostrocaudal sequence of sections are labeled from **(A–D)**. Drawings of coronal sections with major dopamine cell groups illustrating cells that were immunopositive for tyrosine hydroxylase (TH; red) and cholera toxin B (CTb; green) after an injection of CTb into the posterior part of the paraventricular nucleus of the thalamus. The location of TH/CTb double-labeled neurons are shown as black stars. Numbers on the bottom right of each drawing represent the distance from bregma. For abbreviations, see list.

**Figure 5:**
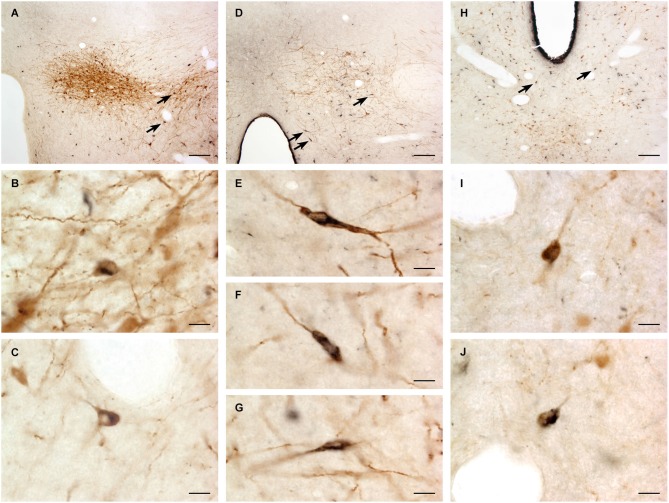
**Examples of double-labeled neurons**. Digital images of the dopamine group A13 **(A)**, A11 **(D)** and A10vr **(H)** showing cholera toxin B labeled neurons (black) and tyrosine hydroxylase immunopositive neurons (brown). The double-labeled neurons (arrows) are also shown at higher magnification for A13 **(B,C)**, A11 **(E–G)** and A10vr **(I–J)**. For abbreviations, see list. Scale bars = 100 μm **(A, D, H)**, 10 μm **(B,C, E–G, I–J)**.

A large number of CTb-labeled neurons were found in many regions of the hypothalamus as previously reported (Otake and Ruggiero, 1995; Li and Kirouac, 2012). Figures 3, 4 show the location of CTb- and TH-labeled neurons in areas of the hypothalamus and midbrain, respectively, following the analysis of an injection of CTb in the pPVT. Based on the large number of CTb-labeled cells in the hypothalamus, it is clear that the injection sites used for the present paper were adequate to examine the sources of the DA fibers in the PVT. Consistent with previous papers, the periaqueductal gray, dorsomedial nucleus of the hypothalamus, and the reticular nucleus of the thalamus were found to be the major sources of inputs to the pPVT (Otake and Ruggiero, 1995; Li and Kirouac, 2012). The distribution of TH-labeled neurons was consistent with previous descriptions of the DA cell groups in the hypothalamus and midbrain (Dahlstroem and Fuxe, 1964; Léger et al., 2010). After close examination of all the cases, we found that the majority of TH/CTb double-labeled neurons were located in the A15 (Figures 3A–C), A13 (Figures 3E,F, 5A–C), and A11 cell groups (Figures 3G,H, 4A, 5D–G). In addition, TH/CTb double-labeled neurons were found scattered in the zona incerta (Figures 3E,F), A12 group (Figure 3E), perifornical hypothalamus (Figures 3D–F), A10vr area (Figures 3H, 4A, 5H–J) and periaqueductal gray (A10dc; Figure 4D). Of note, double-labeled neurons were not found in the VTA (A10), substantia nigra (A9), and the retrorubral field (A8) as shown in Figures 4B,C. Consistent with the fact that the CTb injections involved predominately one side of the PVT, in all cases double-labeled neurons were found bilaterally with an ipsilateral predominance in both the hypothalamus and midbrain (Figures 3, 4). Although the aPVT injections resulted in fewer double-labeled neurons, there were no qualitative differences on the pattern of TH/CTb double-labeled neurons between injections in the aPVT and pPVT. Attempts to quantify the proportion of TH-positive neurons that were CTb-positive were abandoned because these numbers were low and variable across different cases. Nonetheless, it is possible that some double-labeled neurons may have been missed because the dense CTb reaction product found in some neurons may have obscured the TH staining in the same neurons.

There were not many usable “off-target” injection sites in the present study. In fact, the technical issue most often encountered was not of making injections outside the PVT but rather that the tracer did not diffuse out of the micropipette sufficiently to yield an injection site capable of producing retrograde labeling. A couple of injection sites in the paratenial nucleus immediately adjacent to the aPVT produced a large number of retrogradely labeled neurons in the prefrontal cortex but no labeling in the hypothalamus or the midbrain. It should also be pointed out that there was no evidence of double-labeled neurons in the VTA in the cases in which the CTb injection involved the medial most part of the mediodorsal nucleus. This indicates that the PVT and other nuclei immediately adjacent to the PVT do not receive any appreciable innervation from DA neurons in the VTA. Finally, the absence of double-labeled neurons in the VTA, which is known to provide a robust DA innervation of the habenula by way of the fasciculus retroflexus (Gruber et al., 2007), provides further reassurance that the labeling observed was not due to diffusion of CTb outside the PVT or from uptake by damaged fibers traveling through the PVT.

## Discussion

The main finding of the present study is that neurons that are located in the hypothalamus and periaqueductal gray provide the DA fibers to the PVT. Most of the TH/CTb double-labeled neurons were found in the A10vr, A11, A13, A15 DA cell groups of the hypothalamus and A10dc of the periaqueductal gray. In contrast, no evidence was found that the PVT receives projections from DA neurons in the ventral tegmental area (A10, excluding the A10vr and A10dc), substantia nigra (A9), and the retrorubral field (A8). While there are clear differences between rodents and primates in the distribution and density of DA fibers in the thalamus (García-Cabezas et al., 2009), the findings in the present study show that the origin of DA fibers in the dorsal midline thalamus is the same between these two species (Sánchez-González et al., 2005).

Our findings along with the results of other tracing studies in the rat and primate are in stark contrast with another study reporting numerous double-labeled TH neurons in the VTA and retrorubral field following injections of the retrograde tracer Fluorogold in the PVT (Takada et al., 1990). It is likely that the false positives observed by the Takada et al. study may have occurred because Fluorogold was taken up by damaged DA fibers passing through the dorsal midline thalamus on their way to the habenular complex (Gruber et al., 2007). Labeling of fibers of passage is especially problematic when tracers are applied using pressure injections or when using fluorescent tracers like Fluorogold (Aschoff and Holländer, 1982; Dado et al., 1990; Schofield et al., 2007), as was the case with Takada et al. study (Takada et al., 1990). It is also possible that the misidentification of a significant DA projection from the VTA to the PVT may have been the result of large injections of retrograde tracers that involved parts of the mediodorsal nucleus, which has been shown to receive DA fibers from the VTA (Groenewegen, 1988). The lack of evidence for a DA projection from the VTA to the PVT is also in line with the tracing studies showing an absence of retrogradely labeled neurons in the VTA following injections of tracers in the PVT (Cornwall and Phillipson, 1988; Chen and Su, 1990; Li and Kirouac, 2012). Consequently, we conclude that the majority of evidence available indicates that the PVT does not receive an appreciable projection from DA neurons in the VTA.

What is the potential functional significance of DA innervation of the PVT which originates from neurons scattered in the hypothalamus? This is a question that can only be speculated at because of the paucity of information about the function of these DA neurons. One possibility is that this projection may be involved in arousal since a subpopulation of DA neurons in the hypothalamus was shown to be activated in rats that had explored an open field (A11 group) and after sleep deprivation (A11 and A13 groups) (Léger et al., 2010). While this has not been demonstrated, it is possible that the DA cell groups in the hypothalamus have functions related to DA cells in the VTA. As such, the release of DA along with other transmitters like orexins and cocaine- and amphetamine-related peptide (James et al., 2010; Matzeu et al., 2014) could act on the PVT to modulate reward and motivated behavior. In addition, the A13 DA cell group has been shown to project to the central nucleus of the amygdala and the periaqueductal gray matter (Eaton et al., 1994; Messanvi et al., 2013), two key areas involved in defensive behaviors (McNaughton and Corr, 2004). This suggests that the A13 DA group and the PVT may be part of a network involved in the modulation of defensive behaviors. Indeed, recent experimental evidence indicates that the PVT plays a role in fear, anxiety and avoidance. For instance, microinjections of excitatory neuropeptides called orexins in the PVT of rats were found to produce anxiety-like behaviors while blocking of orexin receptors had anxiolytic effects (Li et al., 2009, 2010a,b; Heydendael et al., 2011). In addition, microinjections of orexin antagonists in the PVT were shown to attenuate conditioned place avoidance to morphine withdrawal (Li et al., 2011) and lesions of the pPVT were reported to attenuate a conditioned fear response (Li et al., 2014). Numerous studies have also documented that neurons in the PVT are activated in animals exposed to a variety of stressful/aversive situations (Beck and Fibiger, 1995; Bhatnagar and Dallman, 1998; Bubser and Deutch, 1999; Timofeeva and Richard, 2001) and it is possible that DA signaling may be involved in the activation of the PVT following high levels of arousal. Finally, the A15 DA group has been shown in some species to be associated with neuroendocrine functions including seasonal inhibition of gonadotrophin-releasing hormone (Hileman and Jackson, 1999; Goodman et al., 2010). Consequently, the A15 cell group may influence the PVT in a way that helps coordinate arousal levels and behaviors with the hormonal status of an organism.

It is also interesting to note that the primary DA receptor found in the PVT is the D3 receptor where the expression of this receptor is high compared to many areas of the rat (Stanwood et al., 2000; Haight and Flagel, 2014) and human brain (Rieck et al., 2004). The D3 receptor is of clinical interest because it has been linked to the regulation of the cognitive symptoms of schizophrenia and the motivation to seek drugs in addicted individuals (Gross et al., 2013; Micheli and Heidbreder, 2013). While the potential therapeutic effects of the D3 antagonists have been shown to be mediated by D3 receptors in the nucleus accumbens (Gross et al., 2013; Micheli and Heidbreder, 2013), it is also possible that some of these effects could be mediated by D3 receptors in the PVT. For instance, the PVT has been shown to modulate DA release in the nucleus accumbens (Jones et al., 1989; Parsons et al., 2007; Choi et al., 2012) and D3 antagonists could act at the PVT to down-regulate the enhanced DA release in the nucleus accumbens associated with schizophrenia and drug seeking. In line with that hypothesis, lesions of the PVT were shown to attenuate the conditioned aspects of behavioral sensitization to cocaine possibly through a D3 mediated mechanism in the PVT (Deutch et al., 1998; Young and Deutch, 1998). Blocking of D3 receptors has also been shown to attenuate fear expression in conditioned rats (Swain et al., 2008) and the PVT represents a potential site for this effect.

In summary, the present tracing study shows that DA innervation of the PVT originates from the hypothalamus (A10vr, A11, A13, A15) and periaqueductal gray (A10dc) and not from the midbrain’s A8, A9 and A10 DA cell groups. The type of signal carried by these fibers and functional consequence of DA release in the PVT are not known but the PVT represents a unique site for D3 receptor antagonists to exert some of their pharmacological effects. Future research may identify more specific effects of DA on PVT mediated behaviors.

## Conflict of interest statement

The authors declare that the research was conducted in the absence of any commercial or financial relationships that could be construed as a potential conflict of interest.

## Abbreviations

3V: 3rd ventricle
A8: retrorubral dopamine group
A9: substantia nigra
A10: ventral tegmental area
A10dc: periaqueductal gray matter
A10vr: A10 ventrorostral dopamine group
A11: A11 dopamine group
A13: A13 dopamine group
Aq: aqueduct
CM: central medial thalamic nucleus
cp: cerebral peduncle
DA: dopamine
f: fornix
fr: fasciculus retroflexus
Hb: habenular
IMD: intermediodorsal thalamic nucleus
MD: mediodorsal thalamic nucleus
ml: medial lemniscus
mt: mammillothalamic tract
opt: optic tract
PT: paratenial thalamic nucleus
PVT: paraventricular thalamic nucleus
Rt: reticular thalamic nucleus
sm: stria medullaris of the thalamus
VTA: ventral tegmental area
xscp: decussation sup cereballar ped
ZI: zona incerta.
